# The wicked problem of waste management: An attention-based analysis of stakeholder behaviours

**DOI:** 10.1016/j.jclepro.2021.129200

**Published:** 2021-12-01

**Authors:** Giuseppe Salvia, Nici Zimmermann, Catherine Willan, Joanna Hale, Hellen Gitau, Kanyiva Muindi, Evans Gichana, Mike Davies

**Affiliations:** a The Bartlett Faculty of the Built Environment, University College London, 14 Upper Woburn Place, WC1H 0NN, London, UK; b UCL Institute for Sustainable Resources. Central House, 14 Upper Woburn Place, WC1H 0NN, London, UK; c UCL Centre for Behaviour Change, 1-19 Torrington Place, WC1E 7HB, London, UK; d African Population and Health Research Center (APHRC), P.O. Box 10787-00100, APHRC Campus, Kitisuru, Nairobi, Kenya; e County Government of Kisumu, P.O. Box 2738-40100, Kisumu, Kenya

**Keywords:** Solid waste management (SWM), Attention-based view (ABV), Stakeholder behaviour, Wicked problems, Low-income, Lower-middle-income countries, Sustainability

## Abstract

Surging amounts of waste are reported globally and especially in lower-income countries, with negative consequences for health and the environment. Increasing concern has been raised for the limited progress achieved in practice by diverse sets of policies and programmes. Waste management is a wicked problem characterised by multilayered interdependencies, complex social dynamics and webs of stakeholders. Interactions among these generate unpredictable outcomes that can be missed by decision makers through their understanding and framing of their context. This article aims to identify possible sources of persistent problems by focussing on what captures, shapes and limits the attention of stakeholders and decision-makers, drawing on the attention-based view from organisation theory. The theory describes the process through which issues and opportunities are noticed and how these are translated into actions, by focussing on the influencers at the individual, organisational and context scale. Views on issues and opportunities for waste management were collected in a series of fieldwork activities from 60 participants representing seven main types of stakeholders in the typical lower-middle income Kenyan city of Kisumu. Through a thematic analysis guided by the attention-based view, we identified patterns and misalignment of views, especially between government, community-based organisations and residents, which may contribute to persistent waste problems in Kisumu. Some point to detrimental waste handling practices, from separation to collection and treatment, as the main cause of issues. For others, these practices are due to a poor control of such practices and enforcement of the law. This study's major theoretical contribution is extending the application of attention theory to multi-stakeholder problems and to non-formalized organisations, namely residents and to the new field of waste management. This novel lens contributes a greater understanding of waste issues and their management in Africa that is relevant to policy and future research. By revealing the “wickedness” of the waste problem, we point to the need for a holistic and systems-based policy approach to limit further unintended consequences.

## Highlights

• Attention-based view helps understand multi-stakeholder waste management problems.
• Our analysis highlighted individual, social and contextual factors driving attention.
• Household behaviour and government control are pointed as main source of the issues.
• Misaligned scales in issues and moves across stakeholders must be tackled.
• We rapport waste management wicked problem to multiple actors and agendas.

## Graphical abstract

**Figure:**
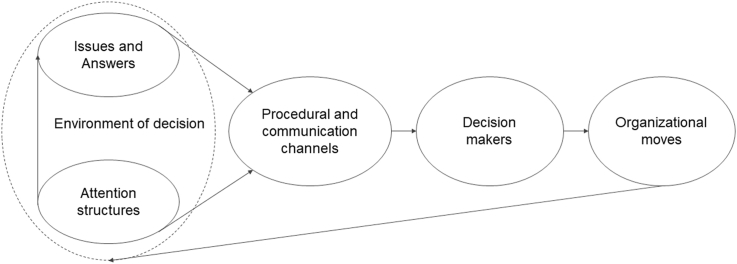

## 1 Introduction

Waste management is a global challenge (Wilson and Velis, 2015) because of the significant fraction of greenhouse gas emissions generated by waste treatment and disposal (Kaza et al., 2018), and a priority to be addressed to ensure sustainable production and consumption (United Nations, 2020). The pressure is acute in low and lower-middle income countries, where growing amounts of waste caused by increased population (Wilson and Velis, 2015), urbanization trends and economic development (Modak et al., 2016) have produced alarming negative impacts, primarily on human health and the environment (Ferronato and Torretta, 2019; Hyman, 2013). Lower-middle income countries account for about a third of the waste generated globally, with sub-Saharan African countries in particular projected to triple the amount of generated waste by 2050 (Kaza et al., 2018). These countries are most affected by ineffective waste management, especially because of a lack of infrastructure, proper management planning as well as insufficient financial resources, technical expertise and public attitude (Srivastava et al., 2015).

Kenya is one of the many countries in sub-Saharan Africa affected by the problem of waste (Kaza et al., 2018). This study focusses on Kisumu, a typical example of a growing city in Kenya, which has experienced significant challenges in relation to insufficient waste management systems (Gutberlet et al., 2017). In Kisumu, and Kenya more broadly, diverse policies have been developed and implemented to address the reduction and optimization of waste management (World Health Organization, 2018). The Kenyan Solid Waste Management Strategy (NEMA, 2015) intends to foster the uptake of efficient technology, yet technological solutions alone are likely to be insufficient to the problems of increased waste, as waste management is driven by multi-dimensional factors (Guerrero et al., 2013).

A recent bill on waste management by the County Government emphasizes the importance of public participation and the collaboration with relevant stakeholders (County Government of Kisumu, 2020); this resonates with the recommendation of previous research indicating that the multidimensional nature of waste management in Kisumu “requires the active participation of all relevant stakeholders including the City Board Management, civil society, NGOs, CBOs, waste private collectors and entrepreneurs” (Sibanda et al., 2017, p. 399). In other comparable contexts, the engagement of multiple stakeholders has been pursued in the past, especially in informal settlements. Public-private partnerships have also been recurrently explored (Ma and Hipel, 2016), on the grounds that public provision of waste management is inferred to yield worse results in countries with lower GDP (Simões and Marques, 2012); nevertheless the engagement of the private sector does not ensure successful results (Simões et al., 2012). Some projects have also engaged residents and waste pickers in collaborative development of basic services with local governments (e.g. Zapata Campos and Zapata, 2013), yet many challenges are faced by these types of projects (Kain et al., 2016).

Previous efforts have attempted to engage a wide set of stakeholders in the development of waste strategies in Kisumu. Nevertheless, both policy and research raise concerns about the limited impact that policies have achieved in practice (Kain et al., 2016). Kain et al. (2016) highlight how a mismatch of views about waste may contribute to the problem. Through analysis of the effects of a plan for waste management in Kisumu, they inferred that policy developers' reframing of waste, from a dirty problem into a resourceful service, was not consistent with the views of other stakeholders, both those directly involved in waste management (such as waste pickers and residents), and those not directly involved (e.g. landlords or residents of some settlements). These other stakeholders did not share the policy developers’ view of change, and some prioritised coping with other difficulties. Ultimately this hindered the anchoring of the Kisumu waste management programme in a fully successful fashion, especially in some informal settlements (Kain et al., 2016).

This study considers how theories of organisational attention could explain mechanisms that drive how stakeholders notice and process changes. Organisations hold understandings of problems, opportunities and the surrounding context which drive their actions towards (sustainable) change. In organisation studies these include collective action frames (Blomsma, 2018) and the institutional logics perspective (Arena et al., 2018; Gregori and Holzmann, 2020). Nevertheless, such understandings are not comprehensive and risk failing to capture critical dynamics. By contrast, theories of organisational attention suggest that organisations are problem-solving entities with limited attention; understanding the behaviour of organisations and their ability to adapt to change requires the understanding of how the attention of their decision makers is distributed and regulated (Ocasio, 2011) for making sense of the environment and its changes (Hoffman and Ocasio, 2011). A multitude of factors within an organisation determine if and how crises are identified, interpreted and addressed, as well as the consequences of the actions enacted (or not) to respond to them (Ocasio, 1997).

A comparative and detailed investigation of how diverse local stakeholders understand the management of waste in Kisumu, and what should be changed, is still missing in our knowledge, despite some appreciable contributions (e.g. Kain et al., 2016). This study addresses that lack. We aim to identify what drives and shapes the attention of decision makers in order to add further insight about the discrepancies among stakeholder views on local waste management reported by Kain et al. (2016). The objective is to find whether and how some of the criticalities and unintended consequences in waste management result from what drives the attention of relevant players, and therefore disparities of what they consider salient. In order to test the alignment of understandings, we engaged stakeholders to represent the diverse sectors involved, i.e. government, industry and trading, community-based and non-governmental organisations, academia, and residents’ associations.

The reminder of the article is structured with a preliminary summary of the Attention-Based View of the organiation (ABV), used to analyse the results of the fieldwork activities (section 2), followed by the methods for data collection and analysis, including a brief description of the case study (section 3). The results (section 4) present two main themes resulting from the analysis: stakeholder views on waste handling practices; and assessments of government's role in these. These themes are discussed by expanding on what constitutes an issue for the stakeholders involved, and the limits in the ways this is addressed, from which we argue that waste management is a ‘wicked problem’ (section 5). The key insights and contributions of the article are summarized in the conclusion (section 6).

## 2 Attention based view (ABV): theory and applications

This study draws on organisational research addressing “the socially structured pattern of attention by decision makers within an organisation” (Ocasio, 1997, p. 188). Diverse elements drive decision makers’ attention, according to a review of the literature (Suzuki, 2017), including organisational goals, strategy and identity; characteristics of decision makers, individual or collective schemas, cognitive models of key decision makers; organisational positions and roles. These elements reflect that “attention is not a unitary concept but a variety of interrelated mechanisms and processes” (Ocasio, 2011, p. 1286). ABV is a theory of organisational decision-making and action developed by Ocasio. Drawing on Simon (1947), Ocasio (2011) provides an explicit treatment of attention to explain organisational behaviour as a situated, variable, multilevel process that combines cognition and structure. Specifically, cognitive processes are engaged at both individual (i.e. the carrier of focussed attention) and social level (i.e. contextually shared understandings and values). Social, economic, and cultural structures operate in the organisation and determine how attention is distributed. For its multilevel approach, ABV is considered a cornerstone breaking engrained assumptions in the field (Kaplan et al., 2001) and it has been used successfully to explain organisational decision-making processes, organisational change and management innovation, amongst others (Ferreira, 2017). However, its application to understanding waste management has been limited to date.

**Fig. 1:**
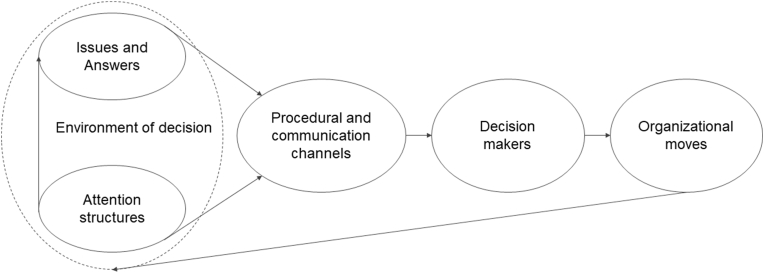
ABV model; simplified version of the original one by Ocasio (1997) representing the process according to which (from left to right) issues and answers in the decision environment are shaped by attention structure and progressively elaborated through procedural and communication channels in order to guide decision makers towards the enactment of organisational moves. The arrows indicate the direction of the influences between the elements of the model.

Ocasio (1997, p. 189, emphasis in original) defines organisational attention as the process of “noticing, encoding, interpreting, and focussing of time and effort by organisational decision-makers on both (a) *issues* (…) and (b) *answers*”. *Issues* indicate the available repertoire of categories for making sense of the environment, which include problems and threats, as well as opportunities; whereas *answers* refer to the available repertoire of action alternatives, including proposals, routines, projects, programs, and procedures (Ocasio, 1997). According to Ocasio's model (Fig. 1), issues and answers are conveyed and distributed into specific procedures and communication channels, i.e. the formal and informal activities, interactions, and communications set up by the organisation to induce decision makers to action; these include meetings, reports and protocols. Attention is situated in these channels and therefore managers' attention is conditioned by the interactions between them (Joseph and Ocasio, 2012).

The distribution of issues and answers into the channels is catalysed by attention structures, i.e. the social, economic, and cultural rules that govern the allocation of time, effort, and attentional focus of organisational actors in their decision-making activities (March and Olsen, cited in Ocasio, 1997). Attention structures include contextual rules about how to interpret and operate in reality; players with their skills, beliefs and values; roles and relations within and outside the organisations; and resources necessary for the organisation to perform activities.

Procedural and communication channels as well as attention structures determine the salience of the issues and answers to be attended to; although potentially confusing in their naming, they introduce concrete actions and context respectively in the decision-making process (Barnett, 2008).

These mechanisms guide decision makers towards the definition of organisational *moves*, i.e. “the myriad of actions undertaken by the firm and its decision-makers in response to or in anticipation of changes in its external and internal environment” (Ocasio, 1997, p. 201).

In this study, we apply Ocasio's theory to explore *issues*, *answers* and *moves* as the focus of our investigation. Issues and answers are of paramount importance, because these two “together constitute the corporation's agenda and are central to adaptation and change” (Joseph and Ocasio, 2012, p. 637). Therefore, they are envisaged here as principal proxies for the identification of critical elements. The exploration of moves is likewise relevant in progressing towards issues and answers as well, because, once enacted, the organisational move becomes part of the environment of decision making, and in turn inputs to the construction of subsequent organisational moves (Ocasio, 1997). Also known as ‘automorphism’, such use of past strategies may institutionalize solutions and therefore gain legitimacy not only within the actant organisation but also in the wider field (Schwartz, 2009).

## 3 Materials and methods

### 3.1 The case study of solid waste management in Kisumu

**Fig. 2:**
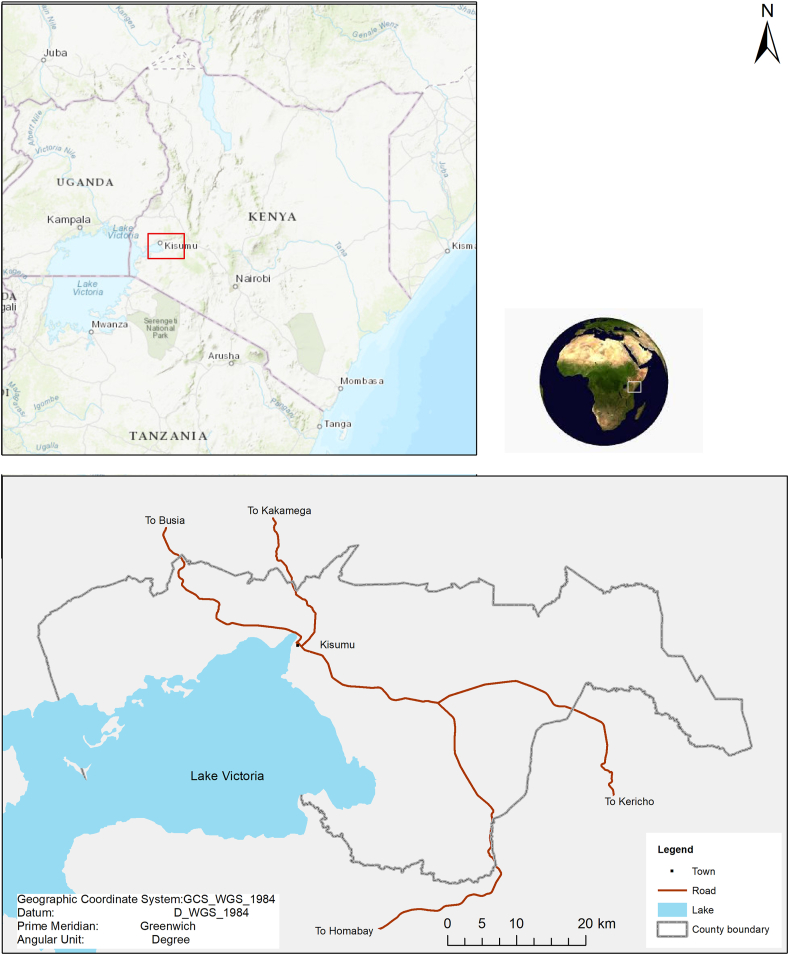
Map of Kisumu county.

This study addresses the issues and solutions envisaged by the stakeholders of waste management in the Kenyan county of Kisumu (Fig. 2), where poor health and the degraded environment are associated with improper disposal of municipal solid waste (County Government of Kisumu, 2019). Population growth, urbanization and lifestyle change, accessibility and illegal dumping are some of the socio-economic and geographical conditions putting pressure on the management of waste for the county, as well as for the wider country (Henry et al., 2006).

The county of Kisumu is inhabited by 1.1 million ca. people (Kenya National Bureau of Statistics, 2019), with an overall population growth trend projected for the next decades (United Nations, 2019). About half of the population resides in urban areas, especially in Kisumu city, which is the third largest city in Kenya (Kenya National Bureau of Statistics, 2019). About 60% of the urban population is estimated to live in slums and peri-urban settlements (United Nations Human Settlements Programme (UN-HABITAT), 2005), which are densely populated areas with limited access to basic services, including piped water (Frediani, 2015), electricity, sanitation, and solid waste management services (Onyango et al., 2013). In the slums, most household waste remains uncollected, mostly because of accessibility and financial constraints (Munala and Moirongo, 2011), and is dumped along roads, alleyways or in vacant lots, leading to appalling conditions (Gutberlet et al., 2017).

Kisumu County generates about 200–450t of solid waste per day, mostly composed of organic material, e.g. food waste (County Government of Kisumu, 2019), in line with other low- and middle-income countries (Modak et al., 2016). Trends of increased waste generation are associated with lifestyles changes (Munala and Moirongo, 2011), possibly in conjunction with urbanization.

The generated waste is handled by both public and private stakeholders. The Kisumu Integrated Solid Waste Management Plan (KISWaMP) combines centralized modes of service provision with grassroots initiatives for expanding the coverage of waste management services to informal settlements where open pits are widely used to manage solid waste (County Government of Kisumu, 2017). Waste is either collected, disposed of in collection stations, dumped or burned. The door-to-door collection is operated by private collectors in affluent neighbourhoods, whereas community-based organisations (CBOs) and non-governmental organisations (NGOs), as well as individual waste scavengers, are mostly operative in the informal settlements. CBOs are groups of individuals who organise themselves to provide waste services in their (usually poor) neighbourhoods; this represents for many an opportunity for both income and a clean environment for the community (Aparcana, 2017).

Waste in the small city centre and the markets is collected by the county government. Only about 20%–40% of the total generated waste is collected for disposal at the city's open landfill (Dianati et al., 2021). Open burning of waste for more than 50 years, only two km from the capital centre (Awuor et al., 2019), at the so-called Kachok dumpsite has raised concerns around insecurity, public health, and environmental degradation (Sibanda et al., 2017). Efforts towards relocating this overflowing dumpsite to a larger site farther away from the city centre have so far not been very successful, mainly because of residents' resistance.

The majority of waste remains uncollected and mostly illegally-disposed, namely openly burnt or dispersed in the environment in garbage heaps and litter everywhere (Munala and Moirongo, 2011), such as alongside roads or on vacant land (Sibanda et al., 2017). Improper waste disposal and management in Kisumu are associated with scarce human and financial resources, poor organisational structures, inadequate legislation and weak enforcement, poor public attitude and low awareness of waste management (County Government of Kisumu, 2017).

### 3.2 Engaging stakeholders across multiple sectors

The study is informed by a set of nine fieldwork activities, including workshops, focus groups and interviews, held in Kisumu in July 2019 with stakeholders of local waste management. Two workshops were held for a variety of stakeholder participants to agree first on a local challenge to be addressed in a bid for funding; the challenge agreed upon was municipal solid waste management. Subsequent focus groups and interviews aimed to collect the views and experiences of stakeholders on this challenge. The participants represented different sectors, specifically civil servants in the county government, academic lecturers, industry and trading associations, CBOs and NGOs, and representatives of the local resident community.

Purposive sampling was used for the invitation of the participants, based on their knowledge of the waste management and sector. Participants were gathered in groups according to their sector (except for the first workshop which covered multiple sectors), with the aim to elicit ‘group thinking’ (Brown, 1999; cited in Robson, 2002) needed to identify patterns of attention distribution and organisational structures within sectors and clusters of organisations. The local government sector was represented by civil servants from departments addressing topics overlapping with waste management, including environment, climate change, energy and urban development. Academics invited were knowledgeable about waste management either through their teaching or research work. A further group of participant mobilizers were individuals from CBOs or NGOs, who reside in Kisumu. A participant from the sugarcane industry – a main industry for the local economy (County Government of Kisumu, 2017) – and two from trading associations attended the focus groups; although limited in number their views complemented the wider picture of waste management. Finally, there were resident association representatives from underserved residential areas, mostly informal settlements in the city. All the participants are operative in the Kisumu county area. With a totalling 60 attendees, the number of participants in each research activity and their sector are summarized in Table 1; abbreviations for fieldwork activities are used to attribute quotes in the Results section, alongside an abbreviation to indicate the specific (male or female) respondent consistently with the transcripts of the activities (e.g. Resident, FR1).

**Table 1:** Number and represented sectors of the participants of each research activity.

Activity number	Activity abbreviation	Sectors represented by the participants	Number of participants (excluding staff)
1	Bid1	Local government, Academia	9
2	Bid2		6
3	CBOs/NGOs	CBOs and NGOs	9
4	Industry1	Industry and trading	2
5	Government1	Local government	10
6	Government2	Local government	8
7	Industry2	Industry and trading	1
8	Academia	Academia	8
9	Residents	Resident associations	7

Each activity started with the participants being invited to introduce themselves and to provide an example of a relevant project on waste management, in which they have been involved. The set of questions for each focus group and interview was designed to inform different streams of the research and including: the goals of the stakeholder groups; barriers and enablers for the achievement of the goals; tensions between organisations and procedures to solve these; decision making processes; evidence use and types; and indicators of success. The number and type of questions were adapted according to the responses and the flow of the conversation in each activity.

The language of all fieldwork activities was English, except for Activity 9 with residents, in which both English and Swahili were used, with local staff members interpreting for the non-Swahili speaking researchers. All the activities were audio recorded with the approval of the participants, all of whom agreed with and signed the informed consent describing the purpose of the study and the research activity; anonymized verbatim transcripts (translated from Swahili where applicable) were provided to the researchers for their analysis informing this study.

### 3.3 Thematic analysis of issues and moves

The transcripts of the fieldwork activities were subjected to the six-phase thematic analysis by Braun and Clarke (2006), a well-established method for identifying, analysing and reporting patterns within data (schema in Table 2). Thematic analysis is widely used in organisational research because it facilitates “the kind of sensitive, nuanced examination of organisational phenomena that qualitative research seeks to achieve” (King and Brooks, 2018, p. 233).

**Table 2:** The six phases of thematic analysis. Reproduced from Braun and Clarke (2006).

Phase	Description of the process
1. Familiarising yourself with your data	Transcribing data (if necessary), reading and re-reading the data, noting down initial ideas.
2. Generating initial codes	Coding interesting features of the data in a systematic fashion across the entire data set, collating data relevant to each code.
3. Searching for themes	Collating codes into potential themes, gathering all data relevant to each potential theme.
4. Reviewing themes	Checking if the themes work in relation to the coded extracts (Level 1) and the entire data set (Level 2), generating a thematic ‘map’ of the analysis.
5. Defining and naming themes	Ongoing analysis to refine the specifics of each theme, and the overall story the analysis tells, generating clear definitions and names for each theme.
6. Producing the report	The final opportunity for analysis. Selection of vivid, compelling extract examples, final analysis of selected extracts, relating back of the analysis to the research question and literature, producing a scholarly report of the analysis.

After having familiarized with the transcripts (Phase 1 in Table 2), the researchers associated codes to reflect the main features described by the text, a process known as coding (Phase 2). Coding followed a hybrid inductive and deductive approach; a preliminary codebook provided deductive (or predetermined) categories reflecting the main premises of the ABV theory for subsequent inductive (or bottom-up) coding, according to which codes are generated to reflect the contents of the data. The coding was performed in NVivo by two coders asynchronously, the work of whom was eventually integrated.

**Fig. 3:**
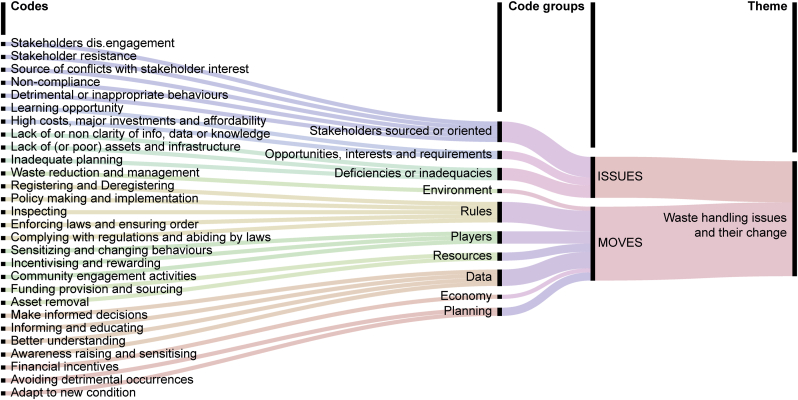
Thematic analysis of the theme ‘waste handling issues and their change’ (on the right). The theme is associated to a set of codes identified in the analysis of the transcripts of the research activities (on the left), which are grouped for convenience (in the centre).

**Fig. 4:**
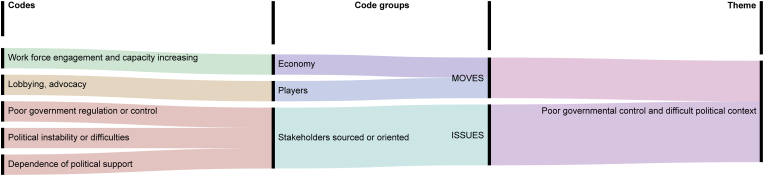
Thematic analysis of the theme ‘poor government control and difficult political context’ (on the right). The theme is derived from a set of codes (on the left), which are grouped for convenience (in the centre).

The set of codes was eventually analysed and reviewed for identification of themes (Phases 3 to 5). The themes presented in the Results are elaborated predominantly from the codes capturing two main elements of the ABV model, i.e. issues and organisational moves, when participants explicitly address waste management. The codes informing the themes are visualized in the Results section in Fig. 3 and Fig. 4, for communication purposes (Phase 6) and transparency (Gioia et al., 2013).

Frequency of references within codes or themes occasionally reported in this article are to be interpreted as a potential beacon of interest for some participants on a specific topic. Reference counts may be affected by multiple conditions and in this qualitative study they are to be considered a potential topic to explore further in data (King and Brooks, 2018, p. 227).

## 4 Results

Two main themes are identified from the analysis: the practice of managing waste, from its generation and separation to collection and treatment; and the practice of ensuring that waste is managed appropriately. The first theme focusses on what constitutes an issue or an opportunity when waste is handled, and how these issues are addressed by the involved stakeholders, such as households, businesses and service providers. The second theme focusses on the ways in which suboptimal, or illegal, waste handling practices are addressed by the government, including expectations and moves for encouraging or enforcing change. Together, the two themes address reciprocal – and often unmet – expectations of appropriate behaviours and roles between two main groups of players (the producers and mangers of waste on the one side, and the government on the other), revealing the different foci and structures driving their attention.

### 4.1 Issues and moves in handling waste

Local waste management is complex, and connected to a wider set of challenges, most notably with health and the environment:

“In Africa we've got all our challenges integrated” (CBOs/NGOs, MR7)

The ways in which waste is handled represent the most recurrent topic, and possibly the priority to be addressed in participants’ description of main issues. The following subsections summarise what participants reported about types of detrimental practices, from waste generation to final treatment, as well as the envisaged solutions for their change. The codes generated from the thematic analysis and associated with this theme are visualized in Fig. 3.

#### 4.1.1 The issues

The most recurrent issues raised across the fieldwork were numerous inadequate or illegal practices of waste handling, and their distribution across many actors. The various stakeholders implicated include: industries with inadequate waste treatment infrastructure (e.g. using burning chambers as incinerators, industry treatment plants); clinics dumping medical waste and even human remains in undesignated areas; waste collectors providing unauthorized services or inappropriately mixing separated waste; and – frequently – households separating their waste improperly, or disposing of it in public spaces (e.g. on roads, in markets). Participants associate significant negative consequences with inadequate waste handling, including both practical limits to the effectiveness of waste management operations, and health implications for operators and the wider population (e.g. because of contaminated water).

The causes of improper waste handling are attributed to multiple reasons and conditions, starting from a lack of the fundamental assets and infrastructure, such as skips, bins and compound facilities in clinics. Likewise, limited financial resources may constrain access to waste management services, namely for clinics or some low-income households, who cannot afford collection fees:

“That is one place skip don't reach there, there are no bins so people just manage their waste the way they think is best for them. So, they burn it.” (Bid2, MR5)

Residents are reported to be uninformed about appropriate ways of handling waste and the consequences of this for health, such as of flooding spreading diseases, because of drainage blocked by dumped waste.

Social norms and routines are also frequently pointed to as significant sources of issues. Improper disposal may hence be rooted in habits. For instance, participants believe that migrants from the countryside to the city are accustomed to disposing of organic waste that is generally biodegradable, and therefore fail to adapt to the need to dispose of non-organic waste in different ways. Moreover, a sense of ownership towards waste management and public spaces plays a role in these behaviours. Shared public understandings of responsibilities have traditionally framed waste as “government's business”, although this is now inconsistent with current regulations:

“if you look at even the waste management regulations it is very clear that it is the responsibility of the generator of that waste to manage it up to the point provided for the government. (…) Anywhere in between here is illegal dumping. So that has been the challenge that for them because also we have very weak infrastructure systems” (Government2, MR1)

In contrast to private houses, which are kept clean, roads and other common spaces are “nobody's land” (CBOs/NGOs, MR4) and waste is often carelessly left there. Traders, for instance, are blamed by those in the government focus group for their attitude to illegal dumping in their areas which may be convenient in the short run, rather than contributing to long-term solutions to the issue. This apparent carelessness provides, and to an extent justifies, jobs to waste collectors:

“<< I will throw this bottle anywhere because the county has employed someone who I assume is supposed to clean the city >>. (…) So, it is more an attitude problem that we are trying to deal with.” (Government2, MR1)

The waste workers are heavily stigmatised (for instance, being referred to as “warthogs”). Some participants want to see this stigma change (Resident MR3). Yet for others, appreciation of these workers' contribution to waste management remains an “impossible attitude” (Resident, MR2):

“[Hotels who refused to pay young task force] think these are just people for ‘takataka’ (Swahili: rubbish collectors)” (Resident, MR1)

Some participants point to meaning and priorities generally attributed to careful ways of handling waste, with a widely shared perception of waste as annoyance rather than a resource – although with notable exceptions reported in the next subsection.

Local policies and regulations as well as initiatives intended to trigger change towards sustainable waste disposal and effective management are in place, and indeed recurrently mentioned especially by the governmental officers, as well as by the industry representatives. Nevertheless, a lack of compliance, and resistance to change, is also frequently reported, notably being more often raised by the governmental sector (Government2 focus group and in the Bid2 workshop session in particular). Residents and representatives of CBOs and NGOs tended to frame these behaviours as disadvantageous (rather than non-compliant) and refer to them to a lesser extent.

#### 4.1.2 The moves

In response to the apparent “illegal” or disadvantageous practices, several possible or enacted moves are reported by the participants, both for a better understanding of the issues, and for triggering change towards more effective waste handling. Local people (e.g. households, landlords, farmers) are often identified, especially by government representatives, as the main sources of issues. This leads to the view that better waste handling should be addressed by discovering the reasons for which they dump, or separate waste improperly, and then by triggering a change in their behaviours.

This behavioural change is proposed through strategies of either encouragement or enforcement. Encouraging strategies include the provision of incentives, e.g. tokens, for reshaping the perceived value of waste and of sorting it. In particular, participants frequently talk of “sensitising” through the provision of information, to raise awareness, and to educate the community and the waste collectors. The suggested means include the engagement of local champions, exhibitions and shows, developing educating platforms, and engaging in practical activities such as clean-ups with the local community. Some of these latter activities have been incentivised and sponsored by governmental organisations, as a proxy for encouraging participation of multiple stakeholders in waste management, and ideally shifting the perception of waste handling away from “the exclusive responsibility of government” (Government2, MR1). Indeed, the governmental sector together with CBOs (and to some extent the residents) mostly advocate sensitization and awareness raising around the importance of a clean environment.

Some participants acknowledge challenges in behavioural change. For instance, they say that extensive time may be required for households to routinise proper waste separation, thus requiring supplementary workforce in separating waste in the meantime, as suggested by a governmental participant. Nevertheless, behavioural change at household level is deemed insufficient by another participant, as the waste management system is not effective:

“(They) are trying to do separation at source and then the same county mixes the waste going to the dumpsite, waste being mixed then again, the waste pickers now do the sorting. You see, it is a bigger challenge.” (CBOs/NGOs, MR3)

This implies that other actors besides households should be encouraged towards better waste handling practices to achieve systemic change. This wider realm of stakeholders to be engaged and connected, most notably waste collectors, is recognized by a participant from the opening workshop:

“(…) you need to look at this holistically, about the issues that are there because you cannot manage waste when you don't have a proper schedule on how you need to collect it, and you cannot have a proper schedule also when you don't have people who are collaborating or cooperating with you to make the environment clean. So, all this boils to one thing that there must be public participation in the entire issue, the government does its role even if they are availing the skips and collection points and whatever, you must also be a co-operator, in terms of from your household, how you are bringing in this waste. The waste collectors, I mean the private, the private waste collectors are very important people, stakeholders in these aspects. Some of them have a proper way of even scheduling their collection either once or twice a week and they know the people, the households where they collect from. So, with time as you try and educate them and talk to them; they will be able to tell you, you can be able to assist us by doing this or that. So, from there you will also be able to learn and get something to know that if this and this is done, we shall succeed from this point of view.” (Bid2, MR1)

Partnerships and collaborations with many stakeholders are often proposed as a move to address the waste once generated, but there is less attention as to what could change behaviours to prevent it arising in the first place.

Networking players is also suggested for maximizing the residual value of waste. For instance, by the collection of organic waste (e.g. from hotels or schools) for use in the production of energy, thereby fostering the local economy. Circular approaches are recommended to supersede landfilling, currently a convenient option which discourages waste separation. Government could make such waste management approaches lucrative and attractive for private entrepreneurs through the incentivizing provision of infrastructure and financial resources, e.g. funding or tax relief for fostering recycling and youth employment, compostable bag production, or a shift to non-burn-technology for medical waste. Incentivizing actions are complemented by discouraging moves, ranging from the removal of services (e.g. skips from where these are abused by waste collectors and clinics), to better regulation, which is favoured by governmental stakeholders, ideally for limiting illegal dumping, inadequate waste separation, or ineffective recycling in industries.

Other types of moves include stronger enforcement, new policies, inspections, and de-registrations (i.e. of private waste collectors from networks or of providers allowed by the public sector when non-compliance is spotted). Inspections are recommended to inform on the misuse of skips and represent an “easier” way to ensure legitimacy in private clinics, with apparently successful results. Similarly, a resident suggests enforcing the principle of shared responsibility within the community, for example, by making citizens surveillant of disposal habits in a circle of close neighbours.

A final area of attention is the enforcement of a ban on the production of some plastic items, which raises conflicting views. The ban is said by an industry representative to have resulted from lobbying pressure on the government by environmental groups and other stakeholders to regulate the market producing waste, especially the high number of water-bottling companies. Banning the production of plastic bottles risks disincentivising recycling, leading to more plastics disposed of in the environment (CBOs/NGOs, MR7). An alternative to the ban is developed by the industry sector in an action plan approved by the government, for the collection of used plastic bottles for remanufacture.

### 4.2 Poor governmental control and the difficult political context

The inadequate practices of waste handling mainly reveal the view of issues and moves from the government perspective. The role of governmental stakeholders is highly relevant to waste management, for they have the legitimacy and ability to define the trajectories of issue resolution. Nevertheless, certain groups of participants often contested the effectiveness of their actions, seeing unmet expectations and thus representing a source of issues to be addressed in waste management. The codes informing this second theme are visualised in Fig. 4.

This issue is raised most frequently by resident representatives and CBOs and NGOs as well as in the initial workshop; few or any references are coded across the other stakeholders. Many expect the government to address waste management better and more intensively by ensuring order through the enforcement of the law, and through implementing policies for change. Issues of order are raised with respect to compliance and illegal actions, to clarifications about procedures to be provided to the community, and to effective collection of separated waste. Policies are reported to be generic, with the resulting risk of amplifying the challenges due to lack of infrastructure.

Urban planners are accused of creating inadequate conditions, failing to deliver on or anticipate, for instance, convenient solutions for the local community; the reconfiguration of urban activities deriving from disruptive interventions; increased pressure on service from population growth over time; or missing designated areas for solid waste especially in informal settlements, which may encourage illegal dumping (Bid2, MR4):

“(W)hat is our planning system? Who is planning for us that I am generating waste in my house, what next should I do with it? should I throw it to my neighbors, should I throw it on the roadside or should I take it somewhere that our urban councils or county governments in a big or smaller way, I am trying to dig out that the planning aspect of it is a major issue that we can be able …. help us address this issue. After generating this waste in my house is there any place that is designated closer to where I am living, where I can take my waste then? Or must I go all the way seven kilometers where Kachok dumpsite is located? So, these are the queries.” (Resident, MR1)

Discussions about unmet expectations, contested actions, and perceived failings reveal possible sources of constraints for the government. These reflect the attentional structures and issues faced and reported by their representatives, including contextual political instabilities, rules of the game for politicians, and salience attributed by decision makers to different stakeholders. Politicians and governmental actors’ personal agendas and priorities are held to drive their moves, with respect to ongoing plans and projects:

“So, in the governor's directive now, because in his manifesto he promised Kisumu people that he will do away with Kachok. And he is already getting rid of Kachok with now timelines.” (Government2, MR4)

Our analysis suggests that two main players attract the attention of the local government and politicians: the national government and the local community.

On the one hand, local government is part of a larger structure with a top-level management at a national scale. The relationship and social norms across representatives of the country's two-tier governmental structure is reported to generate conflicts of interest instead of symbiotic working. Policies intended to bring about sustainable change require the approval of political decision makers, which may result in lobbying, and even bribery and corruption (Government1, MR8).

Likewise, some of the major issues and answers regarding waste management, including the creation of a dumpsite, may be envisaged as opportunities for monetary advantage, said to attract the attention of the political class and higher governmental levels, and thus becoming their interest rather than of the *wananchi*'s (Swahili: citizens'):

“(…) Waste management is not for the poor, it is for the rich.” (Government1, MR3)

On the other hand, the importance for politicians to produce visible and memorable outcomes attracting the attention of the voting local community (e.g. a borehole, a hospital) is a driving force in decision-making processes. This is supported by discussion regarding the allocation of budget to the departments at governmental level. This is observed to be often on the basis of the visibility of the actions (e.g. creating dispensaries or drilling boreholes, rather than cleaning the market), serving as proxies for increasing the chances of re-election for a political candidate.

“Unfortunately, decisions made at this level, a lot of it is driven by politics and politics is about perception. When I build a hospital or dispensary then I stand a chance of people seeing what I have done [… and be re-elected …]. When I clean a market, the traders may have a feeling of that impact. But even then, because it is something recurrent, tomorrow when you come back it is already dirty. So, it doesn't stick in mind. So that dispensary is more long lasting or a road or an ECD center. So, in order of priority they only seem to get the bowl first then whatever remains is given to the rest of us.” (Government2, MR4)

The dynamics of these two poles indicate how the moves operated by government to attract attention of decision makers, or other salient players, may in turn contribute to problems for waste management.

Notably, unlike the set of moves fostering change of practices of waste generators and handlers, there are few actions suggested to address these political issues, constraining rules of the game, procedure and attention structures of local government.

## 5 Discussion

The results of the study confirm how complex the system of waste management is in Kisumu, engaging a number of different stakeholders who pursue a variety of goals through their moves (summary of issues and corresponding moves in Appendix B). This general outcome and several of the specific dynamics resonate with previous studies in this context, most notably with the issues of deprivation, financial scarcity, poor planning (Kain et al., 2016), poor government control and enforcement (County Government of Kisumu, 2019), ambiguity in responsibilities, (County Government of Kisumu, 2019; Gutberlet et al., 2017), poor public attitude to proper waste management and infrastructural inadequacy (County Government of Kisumu, 2019; Gutberlet et al., 2017; Kain et al., 2016; Munala and Moirongo, 2011; Sibanda et al., 2017).

Our thematic analysis identified two contrasting themes, corresponding to an opposite attribution of responsibility and expected actions from other stakeholders (Guerrero et al., 2013); in summary these themes are inadequate waste handling according to the governmental sectors, and ineffective control mostly according to the local community. In our view, these contrasting themes largely emerge through the identification of multi-level drivers of attention enabled by ABV, and which contribute to explaining the discrepancy in views and perceptions of success in policy local implementations.

### 5.1 What constitutes inadequate practices and the limits of sensitisation?

The non-compliance of households in handling waste and resistance to positive change emerged in our first theme, and, consistently with literature (Sibanda et al., 2017), is more recurrently reported by participants from the governmental sector. In our view, this pattern is possibly associated with the area of competence of our participants, and the way success is measured, i.e. the extent to which one of their main outputs (policies) are abided by.

A multitude of moves are proposed or reported as enacted by the participants to change residents' and other waste generators’ behaviours. Raising awareness and “sensitisation” are dominant reported moves by local government, and intended to trigger change in waste handling practices. Nevertheless, our results suggest that information may actually be available to the waste handling actors.

Behavioural science theory and research highlights that these information-provision based approaches are not necessarily sufficient to change behaviour (Gatersleben et al., 2002; Kollmuss and Agyeman, 2002), nor are they the only behaviour change approaches available to policy makers (Michie et al., 2011; West et al., 2019). Information and sensitisation typically aim to change people's understanding and attitudes, but it is well-established that there are gaps between forming an attitude, forming an intention to act, and actually acting (Armitage and Conner, 2000).

A multitude of additional factors affect this practice and characterise the environment of the waste handlers, including rooted habits, financial and infrastructural scarcity, convenience and situated circumstances, perceived ownership and diffused responsibility. Although non-compliant with the law, the ways in which waste is disposed represent accessible solutions to other sets of more relevant or pressing issues.

In order to initiate a desired behaviour, such as sorting waste at source, or to stop an undesired behaviour, such as dumping on the roadside, people need to have sufficient capability (physical and psychological ability, e.g. skills and knowledge), opportunity (features of the physical and social environment, e.g. infrastructure and social norms) and motivation (reflective and automatic processes, e.g. beliefs and habits). These factors form the COM-B model of behaviour (Michie et al., 2011). Stakeholders mentioned examples of each factor as a barrier to proper waste management. For example, a lack of knowledge about how to dispose of waste correctly (capability), a lack of resources such as easy-to-reach bins and skips to dispose of waste (opportunity), and beliefs that waste management is someone else's responsibility (motivation). Successfully changing behaviour in complex systems is likely to require a combination of different intervention types, including education, incentivisation, training, environmental restructuring (Michie et al., 2011), and delivered through multiple policy actions, e.g. fiscal measures, legislation, service provision, communications (Michie et al., 2009).

Nevertheless, evidence about if, and how, households’ environment and behaviours are explored by governmental players to make decisions is limited. Furthermore, consensus on how change towards more sustainable patterns of consumption occurs is not reached in scientific literature; the critiques that some dominant behaviour change approaches receive (e.g. Shove, 2010) reinforce how challenging such a necessary change in the way people frame and carry normality is, and major efforts are required to envisage and develop more robust, effective moves.

### 5.2 Structural determinants of government

Participants often attribute substantial if not sole responsibility for addressing waste management to the government. This interpretation may derive from former governmental arrangements, preceding the establishment of Kenyan Environmental Management and Coordination Act in 1999, which reallocated environmental responsibilities (The Republic of Kenya, 1999). Despite the declared intentions and moves of engaging the wider set of stakeholders, the role of government will likely remain central in setting goals and coordinating actions.

Nevertheless, our analysis showed few suggested moves to change the way government acts (see Appendix B). Possible reasons for this include the potentially more visible nature of issues associated with resident behaviours, and the difficulty of envisaging solutions to possibly perennial problems characterising government attention (e.g. political interests, pleasing voters, two-tier governance and budget constraints). Our results suggest how the capability of county government to trigger change is bounded by structural and procedural determinants across two poles attracting its attention, i.e. the top management and the local community to serve. An issue is salient when it “resonates with and is prioritised by management” (Bundy et al., 2013, p. 353). Nevertheless, the goals of the top management may not necessarily capture the changes important to the voting population. Bansal et al. (2018) elaborate on how organisations may fail to identify latent issues (especially for sustainability) because of the lack of procedural or communication structures to notice them, more specifically because of the inconsistency of scale of the processes that generate the issues.

The issue of scale is important, as in Kisumu the longer-term view of government that elaborates extensive plans and programmes appears temporally misaligned with issues for the local community affecting their shorter-term, or even daily, routines. On one side of the spectrum, the county government pursues strategies intended to meet environmental targets scheduled in five or even 35-year plans; whereas, on the other side of the spectrum, residents report on routinised habits of dealing with cooking waste, market shopping, and corporeal needs. Business, CBOs and NGOs fall in between the previous two, while seeking profits and economic sustainability over the following financial years.

Public participation is required by the constitution (The Republic of Kenya, 1999) and results in a driving force in decision-making processes. The conventional procedural and communication channels deployed by the Kenyan government to collect the issues of the community are reportedly consultation in meetings and engagement in activities. Nevertheless, these means of expression and participation may not necessarily enable latent issues to surface (Sanders, 2000) and therefore the community's underlying problems could easily persist.

The scarcity of financial resources, confirmed in literature (Kain et al., 2016), paired with reported conflicts of interest and corruption when monetary opportunities arise, further restricts the possible pathways for change to be undertaken and may increase chances on unmet expectations from other stakeholders. Therefore, the complex and complicated dynamics characterising the whole set of attentional structures (i.e. players’ salience, organisational roles, sector rules, and resources), as well as the potentially underperforming conventional procedures and communication channels, possibly hinders the current capability for local government alone to trigger the transformational change that waste management in Kisumu requires.

Multiple pathways for government to limit issues and generate benefits towards waste management in low- and middle-income countries are suggested in literature. The participants and literature (Awuor et al., 2019; Gutberlet et al., 2017) often recommend that the government increase its support to solid waste management; in our view, the ABV approach can help understand which structures need to be changed for this. A more structured participatory approach and involvement of stakeholders across the multiple stages of waste management is key, starting from the development of county integrated development plans, most notably residents and CBOs. We also support the recommendation of other studies about the closer and more sustained engagement of the informal sectors in waste collection especially (Wilson et al., 2012). The main benefit usually envisaged is a capillary collection of scattered waste (da Silva et al., 2019; Velis, 2017). The main advantage we foresee in the engagement of this intermediary is the bridging role that the informal sector may play between the narrower scale characterising the local community in waste handling, and the broader one of the government, in ensuring control. Informal waste collectors may inform and interpret between these two players, for their knowledge of both the context (with its social norms and needs) and the policy ambitions and procedures.

Consistent with a wicked problem, this solution necessarily raises new issues, including the increased attentional effort required in relating with more players (Gutberlet et al., 2017), resistance to the formalisation of the engagement, competition with the formal sector (da Silva et al., 2019) and criminal activities (da Silva et al., 2019; Velis, 2017). Nevertheless, it remains to be understood if the benefits will offset the negative consequences.

### 5.3 Systems-thinking for handling the wicked problems of waste management

The results support the view of waste generation and treatment as multidimensional practices (Sibanda et al., 2017), entangled in complex webs of interacting actors (Gutberlet et al., 2017) and situated features, including social norms, political influences, financial availability, let alone infrastructural and technological implementations (Guerrero et al., 2013). Our results indicate how organisational moves and opportunities envisaged by some may result in or be interpreted as issues or threats for others; for instance, convenient disposal in undesignated areas increasing collection pressure, banning of plastics disincentivising recycling, landfilling discouraging separation. The complexity, the social and the endless nature of the causal chains that link stakeholders, and interacting systems contribute to defining waste management as a wicked problem, which is apparently impossible to resolve (Churchman, 1967; Rittel and Webber, 1973).

Systems-based approaches, especially participatory ones, may help towards such wicked problems that are characterised by multiple contrasting views (Vennix, 1999), by limiting the risks of unintended consequences. Some studies adopted a system-based approach to tackle waste management in Kisumu and suggested diverse solutions, namely sensitisation, separation at source, and the formalisation of the informal sector (Gutberlet et al., 2017; Munala and Moirongo, 2011).

Although approaching a complex system by pointing to its individual elements appears reasonable and convenient, we instead propose focussing the discussion on the source that underpins the multitude of issues, i.e. the multiple and varied views of the system across stakeholders, which may generate inconsistencies between expected and actual dynamics in the environment. A systemic study of waste management based on such multiple views could provide a detailed and more comprehensive understanding of dynamics and causal links that result in problematic issues. A lack of a systemic approach to waste management in Kisumu has been previously reported in literature (Gutberlet et al., 2017; Sibanda et al., 2017). Nevertheless, traces of systems thinking emerged evidently in our focus groups, more frequently in governmental representatives, and CBOs and NGOs. This suggests an existing capability to deal with complex systems and linkages. Although the importance of operating holistically is acknowledged by these players, and echoed in the connections across challenges or envisaged solutions, the enacted moves may not necessarily reflect this systemic nature. For instance, whether internal procedures and organisational structure hinders the achievement of intended behavioural change was not raised by the governmental participants. This may be interpreted as inertia, with such structures seen as harder to change than the behaviour of households and other actors. Nevertheless, this interpretation may not reflect the view of the participants and the topic requires additional investigation.

Systems thinking may indeed be hindered by some structures and conditions, such as siloed working within governmental departments, financial constraints limiting the breadth of actions, the political agendas driven by elections; these appear affected by the narrower rather than systemic view of other salient players. In organisational attention literature, specialisation as well as integration of different viewpoints is generally considered important to overcome the limits of individual views and bounded rationality (Vuori and Huy, 2016); this integration should be extended likewise outside the single organisation and across multiple players.

The integration between players faces some challenges. Gutberlet et al. (2017) pointed to weak links between them in Kisumu's informal settings. By drawing on the reflections by Bansal et al. (2018), we suspect a risk of misalignment of scale occurring at their interfaces between stakeholders, which may result in undesirable effects. For instance, waste handling is a highly routinised activity with a short timescale for the local community (daily, weekly, monthly), yet the main proposed or enacted changes especially by government (e.g. more infrastructure and social norm perception) implies change on a long term. In this respect, issues and solutions between the two timescales could be compromised.

In this view, the main question to be addressed when dealing with waste management in Kisumu, and possibly elsewhere, regards the types of tools, strategies, and in particular organisational procedures and communication channels that could enable the link to be made between the multiple levels and players. We know that such procedural and communication channels play a fundamental role in the strategic adaptation of firms, by generating the decision-making patterns necessary to identify opportunities and respond to competitive threats (Joseph and Ocasio, 2012, p. 637). Our analysis suggests that not only intra-organisational, but also inter-stakeholder, procedures and communication channels are important for enacting systemic thinking, attention and moves. How could these channels within the involved organisations be arranged in order to notice, encode and interpret the dynamics occurring at different scales and linking multiple players? How can these surface latent issues across them? These questions require major efforts in future research intending to embrace the ABV approach for systemic change.

Finally, participatory approaches which engage the involved stakeholders in modelling of the system (e.g. Király and Miskolczi, 2019), including residents and intermediaries, are recommended for future studies as a proxy to both capturing the multiplicity of understandings of the issues, and identifying possible leverage for positive change in local waste management.

## 6 Conclusion

We conclude by remarking how waste management in low- and middle-income cities, such as Kisumu, is a wicked problem, for which a solution may not be achieved due to the number and complexity of actors who hold diverse views of the local system. Our study contributed by collecting and analysing the views of a considerable number of stakeholders, representing the variety of sectors engaged across the waste management process and policy making. Our results point to two main issues envisaged by the stakeholders in the persistence of issues: waste handling from separation to treatment in defiance of the law, more frequently pointed to by the government and CBOs; and the poor control over these disadvantageous practices according to others, most notably residents.

The resulting reciprocal blaming, and pointing predominantly to others as the main source of issues, reflects how normality is understood and pursued by the involved actors, who each carry different goals, boundaries of competence and responsibility, ways in which accomplishment is reached and success is measured. In explaining the source of the contrasting views, we infer that the complexity lies not only in the wider waste management system, as acknowledged by the literature, but also in the specific environment of each stakeholder, which is characterised by a multitude of priorities, factors and dynamics attracting their attention.

The research contributes to knowledge in three ways. First, it increases the understanding of waste issues and their management in Africa, where some dynamics, such as costs and efficiency in particular, are under-explored (Simões and Marques, 2012), and specifically in Kisumu, where the perspective and engagement of diverse stakeholders is relevant and urgent (Sibanda et al., 2017). Second, the study explores a novel use of attention theory for complex dynamics with multiple interacting actors towards sustainable change, including non-formalised organisations (e.g. residents); this methodological feature represents the most original contribution in our view, as we are not aware of similar applications of the theory, especially in waste management. Third, the study corroborates the emergent ‘wicked nature’ of waste management (Laura et al., 2020), characterised by problems which are “ill-defined, ambiguous, and contested, and feature multi-layered interdependencies and complex social dynamics” (Termeer et al., 2015, p. 680), and demanding a holistic, system-based approach. The combination of these contributions may inform future methodological approaches to policy making in waste management, which elicit understandings and drivers of attention in multi-stakeholder settings.

We recommend future studies and policies to consider the multiple time and spatial scales characterising the attentional processes for the involved players, and to deliver implementations which address the dynamics at each level in order to plan and anticipate change. Systems-thinking approaches could help in this direction.

The validity and relevance of our results are necessarily constrained by some limits both in the use of the model and in the methodology. The attention-based model was developed and mostly applied for the understanding and representation of formalised organisations, typically firms (Ocasio, 1997). In this study, organisations and stakeholder groups with more variable levels of formalisations than Ocasio's firms were explored and involved.

In this respect, decision-making across these organisations may not necessarily reflect all the mechanisms of the original model. Nevertheless, the fundamental contributions of the model in this study lie in some of its premises and multiple level mechanisms determining the salience of attended issues; and the recognition that enacted moves become part of the future environment of decision and affect the scope of issues which will be attended to.

This paper also suggests and exemplifies how stakeholder groups, in our case residents and CBOs, can be viewed as an entity from the perspective of the ABV. Like an organisation, they are not a homogeneous group, but their attention and decision-making are influenced by established procedural and communication channels and structures. The ability to consider organisations at different levels of formalisation and structure is a necessity for applying ABV to multi-stakeholder problems and provides much basis for future studies. Limits are methodical as well. In the fieldwork activities, the sectors are represented by an uneven number of participants, with a dominant presence of governmental representatives and a minority of representatives of the industrial and trading sectors in particular.

In future research, some of these limits may be resolved or addressed. Although it is not the authors’ aim to generalise the insights of this study, these findings may inform possible critical dynamics and elements of waste management to be further addressed in the future, both locally and in comparable contexts.

## CRediT authorship contribution statement

**Giuseppe Salvia:** Conceptualization, Methodology, Formal analysis, Writing – original draft, Visualization. **Nici Zimmermann:** Conceptualization, Methodology, Investigation, Writing – original draft, Supervision. **Catherine Willan:** Methodology, Formal analysis, Writing – original draft. **Joanna Hale:** Methodology, Investigation, Writing – original draft. **Hellen Gitau:** Investigation, Writing – review & editing, Visualization. **Kanyiva Muindi:** Investigation, Writing – review & editing. **Evans Gichana:** Investigation, Writing – review & editing, Supervision. **Mike Davies:** Investigation, Writing – review & editing, Funding acquisition.

## Declaration of competing interest

The authors declare that they have no known competing financial interests or personal relationships that could have appeared to influence the work reported in this paper.

## Figures and Tables

**Table:** 

#	Questions	Prompts	Research question themes
**PART 1**			
**Signpost**	**In today's activity we would like to find out about your experiences on recent projects related to [TOPIC AREA].**		
**Prompt card**	**Introduction** • **Your name and organisation** • **A project you have worked on** • **Focus of the project** • **Your role in the project**		
**1**	To begin we would like to go around the group and ask you to tell us about yourself and a project you have worked on Please could you introduce yourself and then tell us about your project/work you do *(for government and residents)*, its focus and your role. *Write a one-word name for each project/area of work on the flipchart.*	What do you feel you are responsible for?	Warm up and context of participant's role in decision-making (informal and formal)
**1a**	*If needed, follow up to clarify the focus of the project:* To what extent did the project or does your work consider urban sustainability? To what extent did/does the project/work you do consider health? Is that typical?		Trying to get at the relative importance of e.g. sustainability and health.
**2**	Were/are any of these projects that you talked about connected at all? If so, could you indicate how they are connected and any organisations linking them? *Ask this question to NGOs, Industries, and CBO. It may not apply to residents.* *Ask group to draw and label links on the flip chart to indicate connections*	Did you collaborate with others around the table on any/some/all of these projects? Was there something specific about this/these projects as to why you might have collaborated here but not others? How did the collaboration evolve over time? *Probe to determine decision making process if several projects collaborated towards similar outcome.* *Look at transparency: Clarity of project objectives; equal distribution of risks, benefits, costs etc; practicality of the projects,* e.g. *resource constraints; political acceptability; power etc.*	Same as above and stakeholder dynamics
**2a**	Are there any organisations that are missing on this graphic? *Leave it to the respondent to measure the importance of a partner organisation*	Are they usually involved in such projects?	
Signpost	Next we would like to go into more detail about each project to understand the goals of the stakeholder organisations or groups involved.		
Prompt card	Stakeholders and their goals • Goals of your organisation • Goals of other organisations		
**3**	*Go through the projects one at a time and write responses on a new flipchart sheet:* Regarding the [NAME] project/work, what were the goals of your organisation? Why are these goals important to your organisation? Why? Are these the only goals? What values do your goals reflect? What were the goals of the other organisation(s) involved in the project? Why are these goals important to your organisation? Why? Are these the only goals? What values do your goals reflect? *Repeat the WHY questions until the interviewee talks about underlying values.*	What are the other organisations and stakeholders seeking to achieve? Did any organisations support or object to the proposed project? Why and was there any changes over time? Ask more about the organisations if it is not clear, e.g. is that a community group?	Same as above, organisational identities and stakeholder dynamics
**3a**	Are there any tensions between the different organisations' goals/objectives for the project? Are there any procedures to resolve disagreements if they arise?	Did your organiation have a different goal for what they wanted to get out of the project? Did this create any tension? How did you all manage those different priorities? Did you have a conflict management team which spearheaded conflict resolution?	Stakeholder dynamics, conflict and power
**3b**	What factors have helped to achieve the goals/objectives of the [NAME] project?	Recently or in the past	Barriers to change
**3c**	What factors made it difficult to achieve the goals/objectives of the [NAME] project?	Recently or in the past	Opportunities for change
**4**	Beyond these projects, what are the most important priorities for the city as a whole? *Use flipchart paper* *Be aware of County Development Plans - briefly:* *Nairobi CDP 2018–2022 priorities: built infrastructure; economic growth opportunities; youth, women, disabilities; affordable healthcare; vocational opportunities; food security; governance, public participation; housing; clean energy, safe drinking water, sustainable waste/sanitary services* *Kisumu CDP 2018–2022: Revitalise agriculture; Ensure a healthy population living in a clean and safe environment; Build modern physical infrastructure; Promote skills development; Conserve the environment + lakefront business; Provide housing; Promote sports, culture/arts; Promote vibrant service sector, supported by Sustainable energy).*	Which are the most important goals to achieve for this city?	Trying to get at the relative importance of e.g. sustainability and health.
**BREAK**			
**PART 2**			
**Signpost**	**In the previous part we discussed the goals of the projects you are involved in, and why those goals were important to different stakeholders.** **In the next part we would like to think about how those projects moved from goals to actions, and how decisions were made in that process. We would especially like to understand what factors typically influence decisions.**		
**Prompt card**	**Factors that influence decisions** • **Process of making decisions** • **Factors affecting decisions** • **Information used to inform decisions**		
**5**	When moving from goals to actions, what is the process you or others go through to make decisions?	What is the process you go through? Who is involved? Is this the same or different in different organisations?	Governance and decision-making processes, power Policy agendas, governance, decision-making
**6**	What are the factors affecting decisions being made?	i.e. prompt only if need– structures, people, strategies, resources, organiation, practice norms,	Same as above
**7**	What information do you and your organiation use to inform decisions?	Where does the information come from? Are there alternative policy/development options that were abandoned? Were there any assessments of economic/social/environmental impacts that drove the direction? Did national or other strategies influence the direction?	Same as above and cultures of evidence
**7a**	*If they mentioned research or evidence:* You mentioned the use of research/evidence; how is it used? In case there were any conflicts, did any groups use some research/evidence to back up their position? *If they did not mention the use of evidence:* What is the role of scientific evidence as one of the sources of information?	Try to find out what they actually mean by evidence and research To what extent is research used to inform decisions? What types of research? How is it used?	
**7b**	Is scientific evidence ever one of the sources of information? How is it used? Are other kinds of evidence used as sources of information? How are they used? How do you access such information? (i.e. Do you ‘google search for information, ask a university scientist or an organisation?)	In case there were any conflicts, did any groups use some evidence/information to back up their position? Was there open mindedness among partners, i.e. did partners change their positions?	Perceptions of scientific evidence and its role in decision-making
**7c**	Do you think information can be used more effectively to inform decisions than it is currently used? How?	Are experts sought to provide specialist knowledge? How are they involved in the process and at what time and in what way?	
**BREAK**			
**PART 3**			
**Signpost**	In the previous parts we have discussed goals and decision making in projects related to [TOPIC AREA]. For the final part we would like to talk about the CUSSH project. *Invite participants to read the CUSSH booklet and think about questions or comments. This could be done in pairs.*		
**Prompt card**	**The CUSSH Project** • **Questions and comments** • **Methods and decision-making** • **Sustainability and health goals**		
**8**	Do you have any questions or comments about what you have read? *Take time to make clarifications and explain aspects of the project in more detail.* What are your initial reactions? What is similar or different about our project and the projects we have been discussing?		Perceptions and goals regarding CUSSH
	What are your initial reactions? What is similar or different about our project and the projects we have been discussing?		Perceptions and goals regarding our project
**9**	On our project we are bringing together multiple methods. Are there any methods or combination of methods that you think could inform decisions in Kisumu?	Thinking back to those projects mentioned earlier – how might these methods have helped?	Explore our project approach as a method for providing scientific evidence
**10**	The goals of our project are to achieve transformative changes in the sustainability and the health of cities. What would a transformative change in sustainability look like for Kisumu? What would a transformative change in health look like for Kisumu? *Write on flipchart, leaving space to add specific indicators for each*		
**10a**	What kinds of changes do you think our project could influence?	e.g. changes to stakeholder participation, new local information available e.g. achieving something that could not have otherwise happened, thinking differently about urban health and climate	Perceptions of scientific evidence and its role in decision-making
**11**	Reflecting on what you have read and what we have discussed, do you think our project is too ambitious or large-scale to be useful for Kisumu? If yes, in what ways? If no, do you think its ambitions and scale is about right, or do you think we could be more ambitious and larger scale? If not ambitious enough, what would make it more ambitious?		Possibility for transformational change, facilitators and barriers
**12**	Finally, we want to ask you about how you would determine a general positive development of Kisumu in the long run. What criteria or indicators could be used to measure a successful development in Kisumu? What criteria or indicators could be used to measure a transformative change in sustainability and health in Kisumu? *Add to flipchart, next to what these changes would look like*		This question tests whether the response is consistent with what was said above in terms of attention and goals. It provides measures for transformation at the same time.
**Signpost**	Thank you very much for all of the discussions today. Soon we are going to draw to a close. Before we do, is there anything else that you would like to talk about? *Invite participants to think about their final thoughts and comments then go around the group.*		Wrap-up

**Figure:**
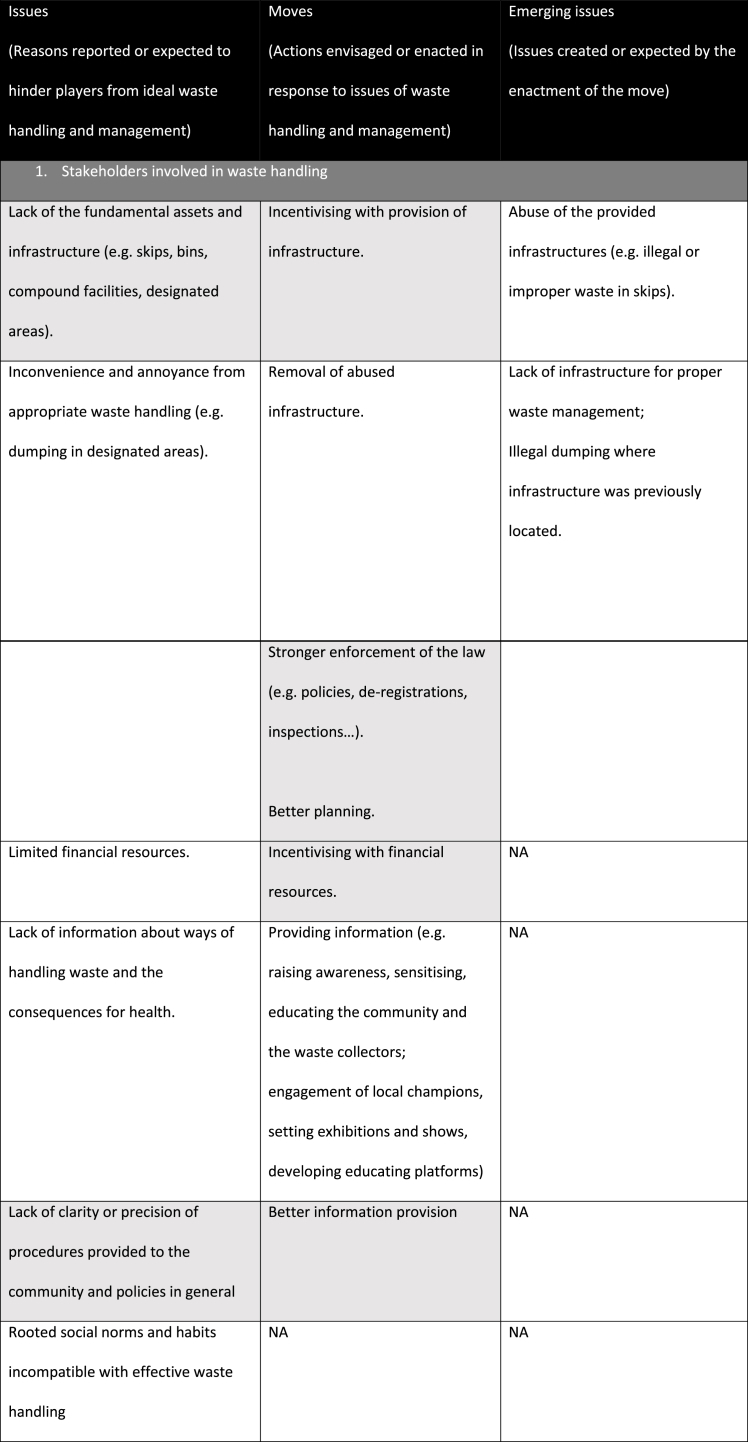

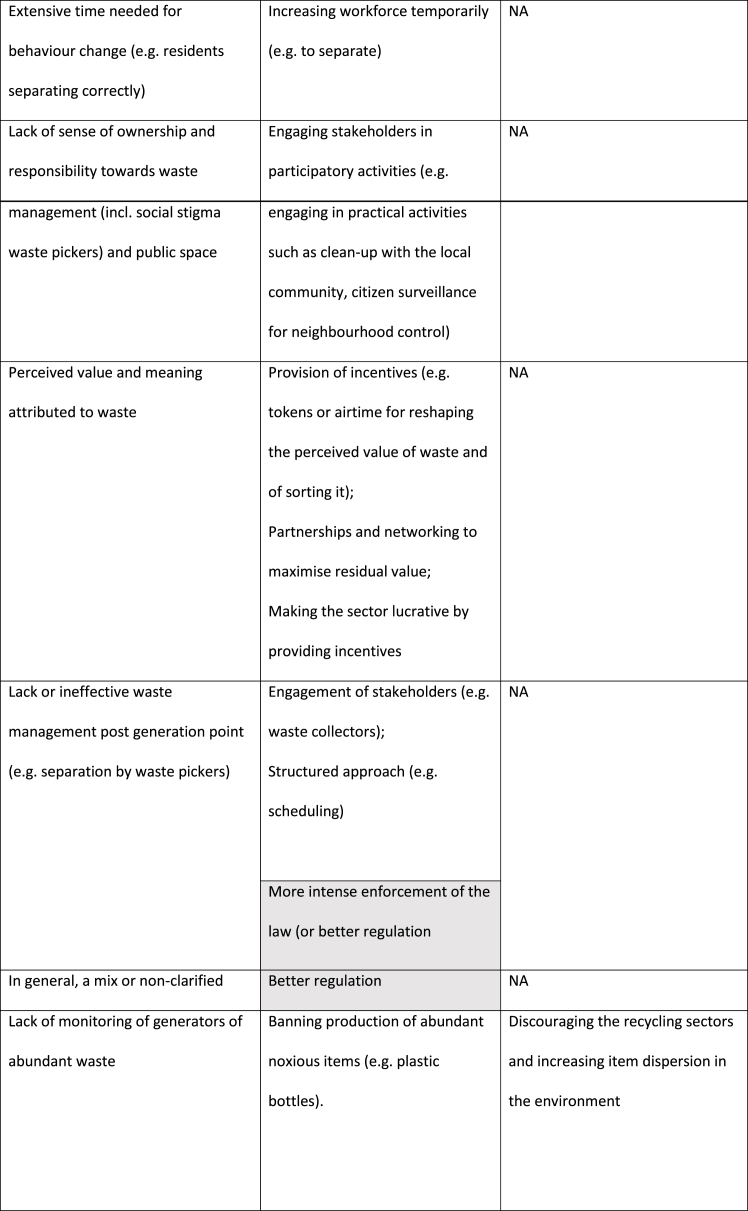

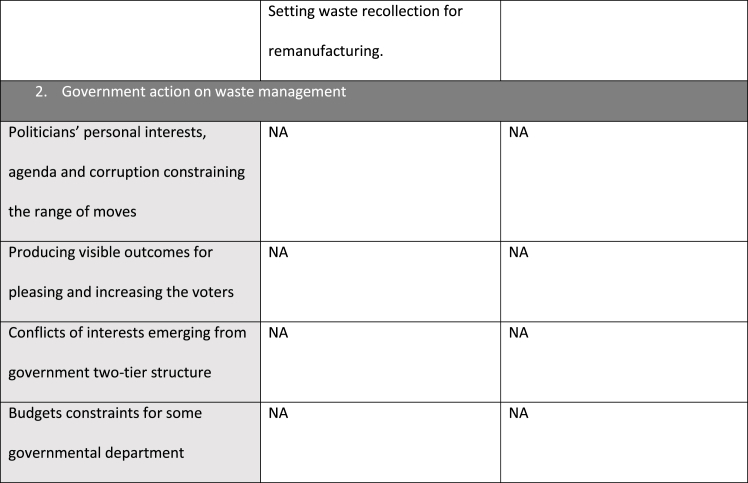


## Appendix A

Guide of the activities, including questions posed to the participants.

## Appendix B

Summary of the results about reported issues, associated moves and new issues to face as either affecting the move or generated by the move. Issues and moves regarding governmental stakeholders are emphasised in light grey.
